# Structural analysis of human NHLRC2, mutations of which are associated with FINCA disease

**DOI:** 10.1371/journal.pone.0202391

**Published:** 2018-08-23

**Authors:** Ekaterina Biterova, Alexander Ignatyev, Johanna Uusimaa, Reetta Hinttala, Lloyd W. Ruddock

**Affiliations:** 1 Faculty of Biochemistry and Molecular Medicine, University of Oulu, Oulu, Finland; 2 Biocenter Oulu, University of Oulu, Oulu, Finland; 3 PEDEGO Research Unit and Medical Research Center Oulu, University of Oulu and Oulu University Hospital, Oulu, Finland; 4 Department of Children and Adolescents, Oulu University Hospital, Oulu, Finland; University of Queensland, AUSTRALIA

## Abstract

NHLRC2 (NHL repeat-containing protein 2) is an essential protein. Mutations of NHLRC2, including Asp148Tyr, have been recently associated with a novel FINCA disease (fibrosis, neurodegeneration, cerebral angiomatosis), which is fatal in early childhood. To gain insight into the mechanisms of action of this essential protein, we determined the crystal structure of the Trx-like and NHL repeat β-propeller domains of human NHLRC2 to a resolution of 2.7 Å. The structure reveals two domains adjacent to each other that form a cleft containing a conserved CCINC motif. A SAXS structure of full-length NHLRC2 reveals that the non-conserved C-terminal domain does not pack against the N-terminal domains. Analysis of the surface properties of the protein identifies an extended negative electrostatic potential in the surface of the cleft formed by the two domains, which likely forms a binding site for a ligand or interaction partner(s). Bioinformatics analysis discovers homologs across a range of eukaryotic and prokaryotic species and conserved residues map mostly to the adjacent surfaces of the Trx-like and β-propeller domains that form the cleft, suggesting both that this forms the potential functional site of NHLRC2 and that the function is conserved across species. Asp148 is located in the Trx-like domain and is not conserved across species. The Asp148Tyr mutation destabilizes the structure of the protein by 2°C. The NHLRC2 structure, the first of any of its homologs, provides an important step towards more focused structure-function studies of this essential protein.

## Introduction

NHLRC2 (NHL repeat containing protein 2) is an essential cytosolic protein of unknown function [1], mutation of which was recently identified in association with FINCA (fibrosis, neurodegeneration, cerebral angiomatosis) disease, a multi-organ condition which is fatal in early childhood [2]. The most prominent features of the disease were severe interstitial pulmonary fibrosis and early-onset, progressive neurodegeneration. The compound heterozygous mutations Asp148Tyr (D148Y) and R201GfsTer6 in NHLRC2 were identified in three children using whole-exome sequencing.

NHLRC2 contains three domains; an N-terminal thioredoxin-like (Trx-like) domain, followed by a six-bladed NHL repeat containing β-propeller domain and then a C-terminal β-stranded domain. The N-terminal Trx-like domain contains a CCINC motif at the position of the CXXC motif, which is characteristic of oxidoreductases and commonly involved in thiol-disulfide exchange. The six-bladed β-propeller domain comprises six NHL repeats, a structural motif originally identified in genes *NCL-1*, *HT2A* and *LIN-41* [3] and shown to often function as a protein-protein interaction module [4].

While mutations in NHLRC2 were associated with a severe disease state, the physiological function of NHLRC2 has not been previously determined. In addition to having an apparent role in early development, NHLRC2 has been suggested to be a novel serum biomarker for Alzheimer´s disease [5] and as having an as yet unresolved role in the regulation of ROS-induced apoptosis [1]. Homozygous deletion of the *NHLRC2* gene leads to an embryonic lethality in mice [2,6] and a recently reported mutation in a conserved region of the β-propeller domain NHLRC2 was shown to cause neural tube defects in Angus cattle [7].

In order to gain the first structural insight of this essential protein of unknown function, whose mutation is associated with a serious disease state in humans, we undertook X-ray crystallographic studies of the protein. Specifically, we crystallized a NHLRC2 fragment containing the human Trx-like and NHL repeat β-propeller domains (aa 1–572) and the NHL repeat β-propeller domain alone (aa 221–572) and determined the crystal structures to 2.7 and 1.75 Å, respectively. Additionally, we obtained a SAXS model of full-length NHLRC2 to provide an insight into the shape and monomeric state of the protein in solution under near-native conditions. Structural studies revealed that the Trx-like domain is connected to the β-propeller via a flexible loop and that the CCINC motif of Trx-like domain is located in close proximity to the binding interface of the β-propeller domain. Bioinformatics analysis allowed us to identify that this protein is not just found in mammals, but a homologue is found across a range of eukaryotic and prokaryotic species. Amino acid sequence-based analysis of NHLRC2 across different species allowed us to map the most conserved residues of the protein to the potential active site of the Trx-like domain and to the spatially adjacent binding surface of the β-propeller. The conservation of residues suggests that NHLRC2 is a redox enzyme with conserved function from across species.

## Materials and methods

### Protein expression and purification

DNA constructs encoding for the full-length NHLRC2 and different NHLRC2 fragments were prepared as described previously and the corresponding proteins were expressed and purified as described [2]. Selenomethionine substituted NHLRC2 (221–572) was produced by inhibition of methionine biosynthesis method [8] and purified similarly to native protein. For crystallization, NHLRC2 (221–572) and (1–572) fragments were transferred to 20 mM BisTris pH 6.5, 100 mM NaCl, 2 mM DTT, concentrated to 8 mg/ml, aliquoted and flash-frozen in liquid N_2_.

### Crystallization

Crystallization screening was performed by sitting-drop vapour-diffusion method in a 96-well plate format using TTP Labtech’s Mosquito LCP nanodispenser and commercially available screens, JCSG-*plus*™, PACT *premier*™ and MIDAS*plus*™ (Molecular Dimensions) and SaltRx (Hampton Research) in the Biocenter Oulu core facilities. Initial plate crystals appeared after 12 hours in JCSG-*plus*™ condition, 0.2 M Li_2_SO_4_, 0.1 M sodium acetate pH 4.5, 50% w/v PEG 400. The crystals were used as seeds in a microseed matrix screening (MMS) experiment [9] with JCSG-*plus*™ screen. Briefly, the initial crystals were crushed in a crystallization drop, suspended into 20 μl of mother liquor in a tube containing the Seed Bead™ (Hampton Research) and vortexed for 30–60 sec. In a MMS experiment, 20 nl of seed solution was combined with 80 nl of reservoir solution and added to 100 nl of protein solution in a crystallization drop. Single diffraction quality crystals were obtained in 0.2 M Li_2_SO_4_, 0.1 M BisTris pH 5.5, 25% w/v PEG 3350. Crystals were briefly incubated in a cryosolution containing 0.2 M Li_2_SO_4_, 0.1 M BisTris pH 5.5, 30% w/v PEG 3350 and flash-frozen in liquid nitrogen. The crystals of selenomethionine-substituted protein were obtained in a similar MMS experiment in 0.14 M CaCl_2_, 0.07 M sodium acetate pH 4.6, 14% 2-propanol, 30% glycerol. Crystals were flash-frozen in liquid nitrogen and stored -70°C.

Crystals of a longer fragment of NHLRC2 (1–572) were obtained in a MMS experiment using crystal seeds of the NHLRC2 (221–572) fragment and JCSG-*plus*™ and PACT *premier*™ crystallization screens. Most crystal hits were obtained in PACT *premier*™ screen and the best-looking single crystals were seeded in a refinement screen designed around the selected conditions. Crystals suitable for diffraction experiments were obtained in 0.1 M BisTris Propane pH 6.5, 0.2 M K/Na Tartrate, 18% PEG 3350. Crystals were cryoprotected in a solution containing 0.1 M BisTris Propane pH 6.5, 0.2 M K/Na Tartrate, 30% PEG 3350 and flash-frozen in liquid nitrogen.

### Data collection and processing

X-ray diffraction data for native and selenomethionine-substituted NHLRC2 (221–572) crystals were collected at European Synchrotron Radiation Facility, Grenoble, France, at ID30B equipped with Pilatus 6M-F detector. Native and anomalous data sets were collected at a wavelength of 0.9790 Å. 360 degrees of anomalous peak data and 166 degrees of high-resolution native data were collected with a 0.15° oscillation angle. X-ray diffraction data for the crystals of a longer NHLRC2 (1–572) fragment were collected at ID30A-3 equipped with an Eiger X 4 M detector at a wavelength of 0.9677 Å. 240 degrees of data were collected at oscillation angle of 0.2°. X-ray data were processed using XDS [10]. Data collection and refinement statistics for the crystal structures are presented in Table 1.

**Table 1:** Data collection and refinement statistics.

	NHLRC2 (221–572) SeMet	NHLRC2 (221–572)	NHLRC2 (1–572)
Data collection			
Wavelength (Å)	0.9791	0.9686	0.9677
Resolution range (Å)	37.8–2.8 (2.9–2.8)	46.3–1.75 (1.81–1.75)	45.8–2.7 (2.79–2.7)
Space group	P 1 21 1	P 1 21 1	P 1 21 1
Unit cell dimensions			
a, b, c (Å)	52.6, 45.3, 70.2	52.6, 46.5, 70.3	103.3,103.8, 115
α, β, γ (°)	90, 102.62, 90	90, 102.9, 90,	90, 100.7, 90
Total reflections	50002	101580	306899
Unique reflections	7878	32606	65003
Multiplicity	6.3 (4.7)	3.1 (2.8)	4.7 (4.9)
Completeness (%)	96.5 (84.3)	96.1 (80.4)	98.9 (99.7)
Anomalous multiplicity	3.4 (2.7)		
Anomalous completeness (%)	93.9 (75.5)		
Mean I/σI	18.62 (5.86)	15.8 (3.27)	9 (1.27)
Wilson B-factor	34.26	17.23	60.1
R_pim_	0.044 (0.14)	0.03 (0.17)	0.068 (0.51)
CC_1/2_	0.99 (0.92)	0.99 (0.92)	0.99 (0.54)
Refinement			
R_work_/ R_free_ (%)		15.3/18.8 (18.2/23.9)	24.7/28.5 (34.6/37.6)
Number of non-hydrogen atoms		2989	17055
macromolecules		2669	17018
solvent		320	37
Protein residues		349	2194
RMS bonds (Å)		0.01	0.01
RMS angles (°)		1.3	1.6
Ramachandran favored (%)		98.26	95.62
Ramachandran allowed (%)		1.74	4.15
Ramachandran outliers (%)		0.0	0.23
Rotamer outliers (%)		0.0	3.9
Clashscore		2.07	0.83
Average B-factor (Å^2^)		21.87	64.71
macromolecules		20.84	64.77
solvent		30.45	36.08

Statistics for the highest-resolution shell are shown in parentheses.

### Structure determination and refinement

The structure of NHLRC2 (221–572) was determined by SAD method using AutoSol Wizard in the PHENIX Software suite [11]. Seven selenomethionine sites were identified by HYSS with a figure of merit (FOM) 0.4 and experimental phases were calculated by Phaser [12]. Density modification was carried out by RESOLVE [13] and an initial interpretable electron density map was obtained. A partial model, composed of a poly-alanine representation of the core β-strands, was obtained by a combination of the PHENIX AutoBuild wizard [14] and manual model building. A higher resolution structure of the NHLRC2 β-propeller domain was determined by molecular replacement using the initial partial model as a search probe and the program Phaser in the PHENIX suite. Approximately 70% of the model was built by the PHENIX AutoBuild wizard and missing regions of the molecule and loops were built manually in Coot [15]. Iterative rounds of refinement were performed in phenix.refine [16].

The crystal structure of a longer fragment of NHLRC2 (1–572) was determined by Phaser within the PHENIX suite using the structure of NHLRC2 β-propeller domain and the thioredoxin-like domain of DipZ (PDB code 2HYX) [17], separately, as search models. Briefly, firstly four chains of β-propeller were found by Phaser followed by the placement of three chains of Trx-like domain. Automatic placement of the fourth Trx-like chain by Phaser failed and central β-sheet and two longest helices were fitted manually followed by iterative rounds of manual model building and refinement. Iterative rounds of model building and refinement were performed in Coot and phenix.refine and REFMAC5 [18], respectively. The analysis of NHLRC2 structure was performed using chain A, for which the electron density quality was marginally higher than for the others.

Model quality was assessed using MolProbity [19]. Figures were prepared in UCSF Chimera [20].

### Small-angle X-ray scattering

The SAXS data were collected on beamline B21 at Diamond Light Source. Scattering data were collected in multiple exposures (60 frames) for each protein concentrations (1.25, 2.5, 5 mg/ml) in 20 mM BisTris pH 6.5, 100 mM NaCl, 2 mM DTT buffer. Solvent scattering from the corresponding buffer was measured before and after each sample, and the average background scattering was subtracted. Programs from the ATSAS package [21] was used for data analysis. PRIMUS [22] was utilized for SAXS data processing and analysis, including R_g_ determination by the Guinier method. The real space radius of gyration (R_g_) and maximum particle dimension (D_max_) were evaluated using GNOM [23], which was also used to calculate the distance distribution functions P(r). 12 independent *ab initio* models of the full-length NHLRC2 were built using DAMMIF [24] and averaged with DAMAVER [25]. The theoretical scattering intensities of the NHLRC2 crystal structure (9–572) were calculated using CRYSOL [26] and the superposition of the DAMAVER generated envelope with the respective crystal structure was performed with the SUPCOMB program of the ATSAS package.

### Size exclusion chromatography multi-angle light scattering (SEC-MALS)

Fragments of NHLRC2 containing the Trx-like and β-propeller domains (1–575) and the C-terminal domain (587–726) for SEC-MALS analysis were prepared by cleavage of the full-length protein with endoproteinase GluC (Roche) at 1:1000 (w/w) ratio overnight at room temperature. The formation of peptide fragments of the correct length was confirmed by mass spectrometry. Purified Trx-like domain with a linker region and β-propeller domain were mixed in equimolar ratio and incubated for 3 hours at room temperature. Full-length NHLRC2, the Trx-like domain, the β-propeller domain, an equimolar mixture of the Trx-like domain and the β-propeller domain, and full-length NHLRC2 after GluC treatment were sequentially loaded onto a Superdex 200 Increase 10/300 size exclusion column (GE Healthcare) pre-equilibrated with 20 mM BisTris pH 6.5, 150 mM NaCl, 2 mM DTT. The eluted samples first passed through a miniDAWN™ TREOS multi-angle light scattering detector (Wyatt technology) and then through a RID-10A refractometer (Shimadzu). The data were analyzed using ASTRA software (Wyatt Technology).

### Biophysical protein analysis

The folded state of purified full-length wild-type NHLRC2 and D148Y mutant NHLRC2 were studied by CD spectroscopy. Proteins were diluted to 0.1 mg/ml in a buffer containing 10 mM sodium phosphate at pH 7.4. CD spectra were measured using a Chirascan Plus instrument and analysed by Pro-Data Viewer software (Applied Photophysics). The melting temperature and melting properties of the purified full-length wild-type NHLRC2 and D148Y mutant NHLRC2 were studied by Thermofluor assay. A range of protein concentrations was tested and 5 μg of wild-type NHLRC2 or D148Y mutant NHLRC2 was chosen as giving the best signal:noise ratio. The reaction was performed in a 96 well plate format in a total volume of 25 μl in 50 mM BisTris pH 6.5, 200 mM NaCl, with 5 μg of protein and 5x SYPRO Orange Protein Gel Stain (Sigma-Aldrich). The buffer with SYPRO was used as a negative control. The reaction was carried out in 3 replicates using Applied Biosystems 7500 Real Time PCR instrument, during which the protein samples were heated from 21 to 90°C with 1°C increment steps and the fluorescence recorded. The melting temperature (T_m_) was calculated as a midpoint of unfolding transition and confirmed by examining of the derivative of rate of change versus temperature.

### PDB and SASBDB accession numbers

Coordinates and structure factors for NHLRC2 (9–572) and NHLRC2 (221–572) have been deposited in the PDB with accession codes 6GC1 and 6G7W. SAXS data and structural model have been deposited in the SASBDB with accession code SASDD86.

## Results and discussion

### Structure of NHLRC2

**Fig 1:**
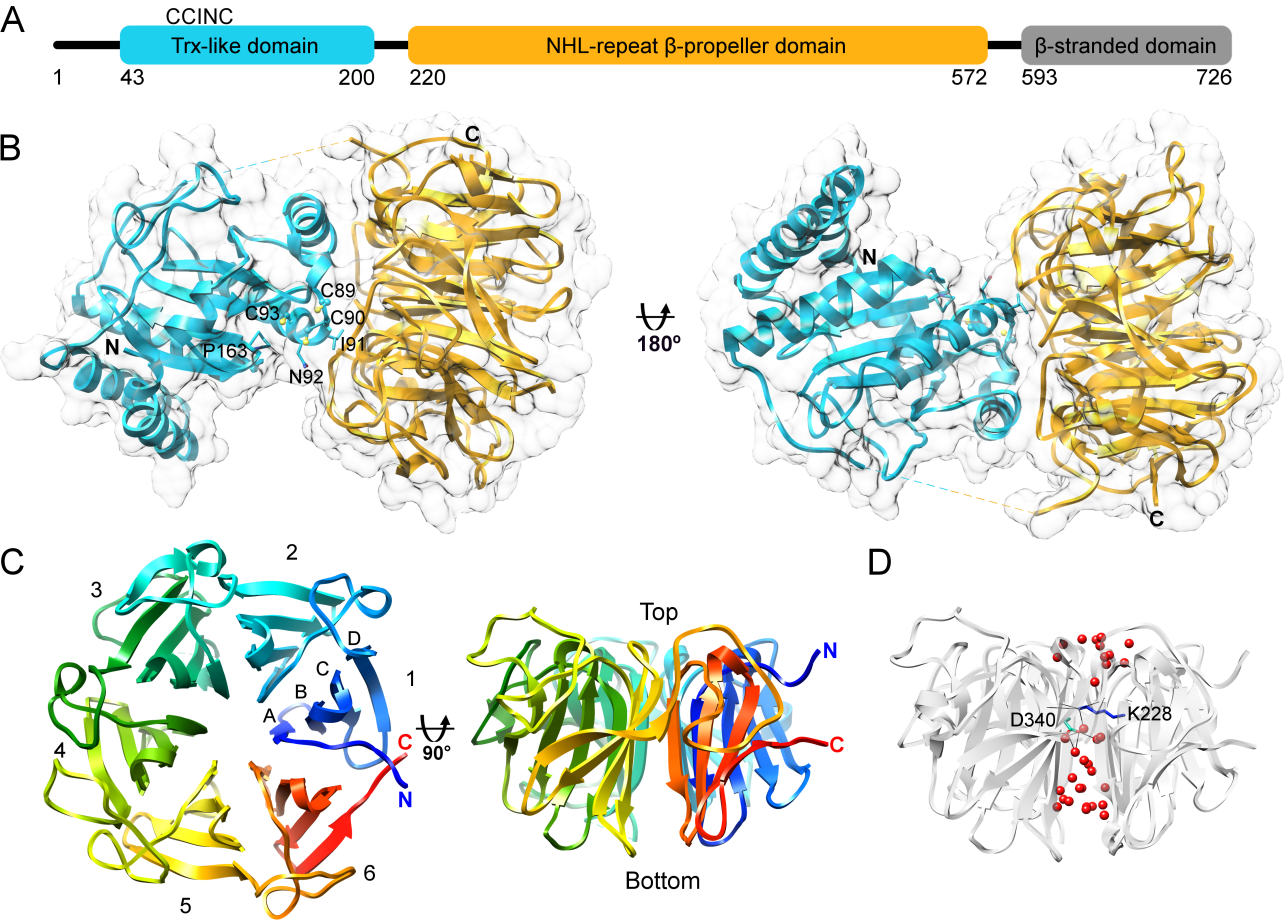
Human NHLRC2 structure. (A) Schematic representation of the domain architecture of human NHLRC2. (B) Ribbon and surface representation of NHLRC2 (9–572) crystal structure with the Trx-like domain colored in cyan and the β-propeller colored in gold. Residues of CCINC motif are shown. Cysteine residues are shown in ball and stick representation and neighboring residues are shown in stick representation. The missing loop connecting the two domains is shown with a dashed line. (C) Two views of NHLRC2 β-propeller are related by 90° rotation around x-axis. Blades are indicated by numbers and strand location is indicated by capital letters. (D) The solvent channel inside of the β-propeller domain contains water molecules (red) and is disrupted by Lys-228 (blue) and Asp-340 (cyan) forming hydrogen bonds with waters and backbone groups of adjacent blades.

Prior to X-ray crystallographic experiments, human NHLRC2 was extensively characterized using bioinformatics methods, including UniProt [27], PSIPRED [28], JPRED [29], and Phyre2 [30], to define the best possible domain borders to aid protein crystallization. NHLRC2 is a 726-amino acid protein and contains a N-terminal Trx-like domain (aa 1–210), followed by a six-blade β-propeller domain (aa 221–572) and then a C-terminal β-stranded region (aa 593–726) (Fig 1A). DISOPRED2 [31] analysis revealed that the C-terminal β-stranded domain is likely to be more disordered and flexible than the rest of the NHLRC2 molecule and is connected to the β-propeller by highly flexible loop. Consistent with this all attempts to crystallize the full-length human NHLRC2 failed despite extensive crystallization screening and seeding experiments. We were able to obtain well diffracting crystals of the isolated β-propeller domain (aa 221–572), and of a longer fragment of NHLRC2 comprising the Trx-like and β-propeller domains (aa 1–572).

The crystal structure of the β-propeller domain was solved by single-wavelength anomalous dispersion method using selenomethionine substituted protein. The crystals of native β-propeller domain diffracted to 1.75 Å, belong to a space group P2_1_ and contain one molecule of the β-propeller in the asymmetric unit. Given the high resolution of the data, the electron density for the main-chain is complete and side chain residues are clearly visible, except for a short fragment between residues 346 and 352 where the electron density is missing.

Crystals of the longer NHLRC2 fragment (aa 1–572) diffracted to 2.7 Å and also belong to space group P2_1_ but contain four protein molecules in the asymmetric unit. The crystal structure of NHLRC2 (aa 1–572) was solved by molecular replacement using the previously determined structure of the β-propeller domain and Trx-like domain of DipZ from *M*. *tuberculosis* (PDB code 2HYX) [17], separately, as search probes. In general, the obtained electron density is of sufficient quality for the main chain and side chain residues for all four protein molecules. However, in each molecule the electron density for first 9 amino acid residues, the loop connecting the Trx-like domain and β-propeller domain (212–219) and the fragment between residues 345 and 354 were either missing or of insufficient quality to allow the chain tracing, suggesting flexibility of these regions.

As expected from the sequence analysis, the structure of the longer NHLRC2 fragment can be clearly separated into two domains (Fig 1B). From amino acid sequence analysis, the Trx-like domain of NHLRC2 classifies to the Trx8 family and is part of the eukaryotic disulfide isomerase-like protein DipZ-like subfamily and plant TlpA-like subfamily of the thioredoxin-fold containing proteins [32]. Consistent with this the Trx-like domain of NHLRC2 displays the common features of a thioredoxin fold, containing a central mixed β-sheet surrounded by three α-helices, along with a Trx8 family specific 26 amino acid insertion comprising an extra β-strand and 2 short α-helices inserted after the second β-strand of the thioredoxin-fold. There are also two additional N-terminal α-helices which fold over the Trx-like domain of NHLRC2 in the crystal structure, forming an N-terminal protrusion.

The CXXC motif or CCINC in NHLRC2 (residues 89–93) is located at the N-terminus of α1 of the thioredoxin-fold, with Cys-89 and Cys-90 protruding from the protein surface (Fig 1B). Together with inserted helices of the Trx-like domain, the coil region containing the CCINC motif is placed the closest to the β-propeller domain and involved in hydrogen bond interactions with it. All cysteines of the CCINC motif are found in the reduced state. A functionally important and highly conserved *cis*-proline (Pro-163 in NHLRC2), which is essential for the maintenance of the structural integrity of the active site of thioredoxin family proteins, is located juxtaposed to the CCINC motif.

The β-propeller domain of NHLRC2 comprises six blades containing four twisted antiparallel β-strands, labeled A–D from the inside to the outside of the molecule, arrayed around a central pseudo-sixfold axis (Fig 1C). The structure also contains six short 3_10_ helices. According to convention, the top face of the β-propeller, a ‘cup’, is defined as the surface that contains the loops connecting strands B–C and D–A, and commonly encompasses a binding site for an interaction partner, a ligand molecule or an active site. The ‘cup’ of the NHLRC2 β-propeller is located at the interface with the Trx-like domain. One interesting feature of the NHLRC2 β-propeller domain is the presence of long extended coil segments on the top or ‘cup’ of the molecule, connecting the C-terminal β-strand of one propeller blade to the N-terminal β-strand of the next blade. In contrast to other β-propeller proteins, the NHLRC2 β-propeller is not closed by a so called ‘Velcro strap’ [33] which is the insertion of the first N-terminal β-strand into the last propeller blade framework. Instead, N- and C-terminal β-strands even though located in close proximity to each other, belong to separate β-propeller blades.

The NHLRC2 β-propeller domain comprises 6 NHL repeats (212–254, 265–307, 335–369, 409–439, 461–505 and 518–562) which are found in each of six blades of the β-propeller and correspond to β-strands A, B and C. The NHL repeats contribute to the high internal symmetry of the β-propeller domain. The β-blades superimpose with an average RMSD ~1.25 Å and primarily diverge in β-strand D and the loops connecting β-strands and sequential blades of the domain (Panel A in S1 Fig). A structure-based alignment of the blade sequences does not reveal a high level of sequence identity between the blades or separate NHL repeats (Panel B in S1 Fig). However, it contains a common motif located on β-strand B and described previously in [34], XXX#§, in which three hydrophobic residues (XXX) are followed by a small residue (#; Ala) and a hydrophilic residue (§; Asp or in blade 3 Met). Conserved prolines are located prior to β-strand A in each blade and a conserved glycine at the end of β-strand D in blades 1–5, allowing tight turns between regular secondary structure elements.

The central pore of the β-propeller domain of NHLRC2 is approximately 12 Å in diameter and contains a number of water molecules (Fig 1D). The continuous water channel is disrupted by hydrogen bonds which Lys-228 and Asp-340 form with water and backbone groups of adjacent blades.

Both Trx-like and β-propeller domains are in close proximity to each other (Fig 1B), with the interaction between them being primarily via a limited hydrogen bond network. This combined with the flexible linker region, suggests they could move relative to each other. As the interaction involves regions around the active site motif of the Trx-like domain and the ‘cup’ of the β-propeller mobility of these domains with respect to each other has possible functional implications.

**Fig 2:**
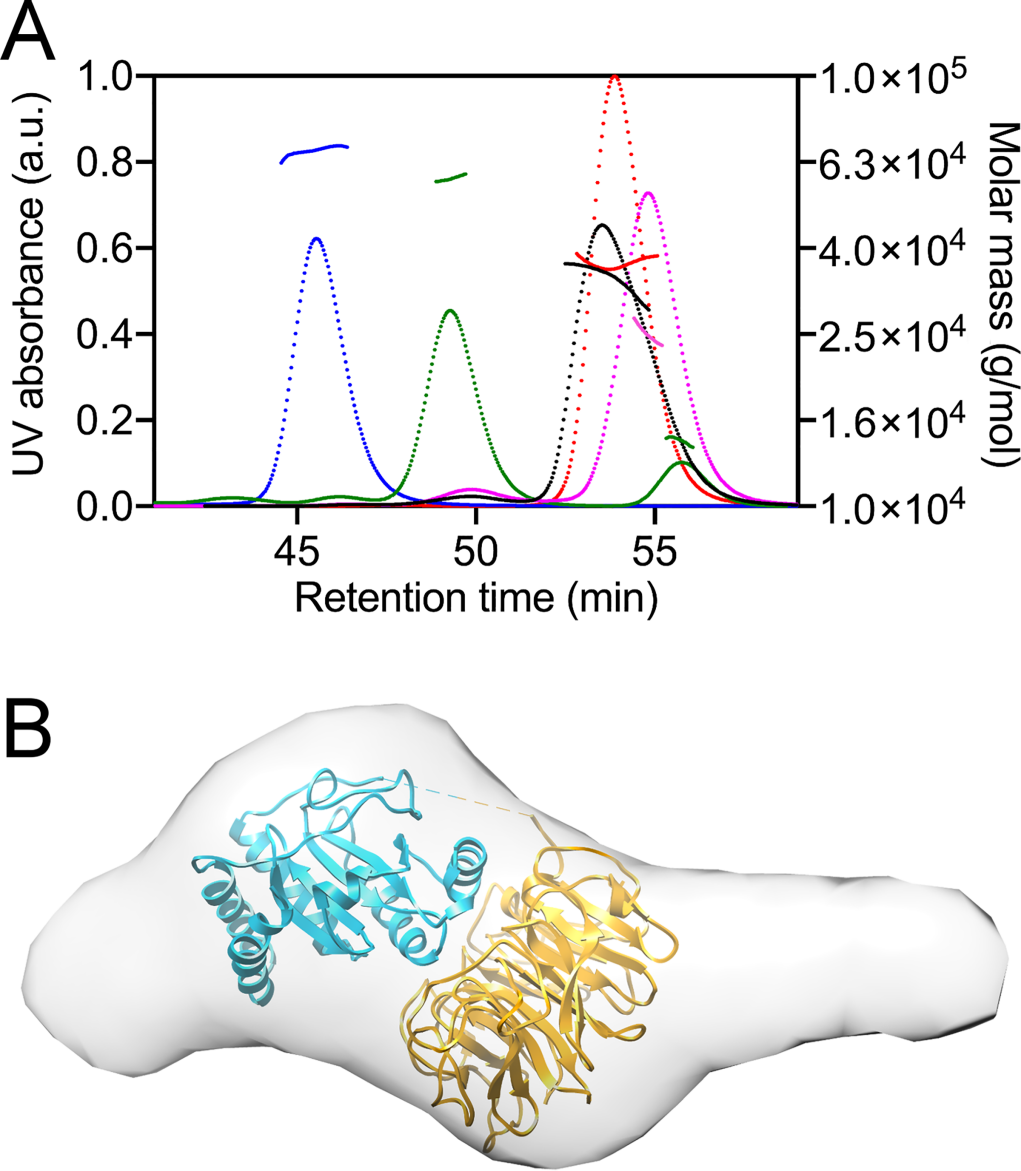
SEC-MALS analysis of NHLRC2 domains interactions and low resolution structure of NHLRC2 determined by SAXS. (A) SEC-MALS traces (with superimposed calculated molar mass traces) of the Trx-like domain (pink, 25.1 kDa; calculated MW 25.8 kDa), the β-propeller domain (red, 35.7 kDa; calculated MW 39 kDa), the full-length NHLRC2 (blue, 69.4 kDa; calculated MW 80.5), NHLRC2 treated with proteinase GluC (green, 59 kDa and 15 kDa; calculated MW 64.1 kDa and 15.3 kDa, respectively) and co-incubated Trx-like and β-propeller domains (black, 35.2–24.8 kDa). (B) The *ab initio* model of the full-length NHLRC2 was reconstructed using DAMMIF and shown is the averaged filtered shape from DAMFILT. The model is superimposed with NHLRC2 (9–572) crystal structure.

The ability of the three domains of NHLRC2 to interact with each other was assessed by size exclusion chromatography linked to multi-angle light scattering (SEC-MALS) (Fig 2A). The purified Trx-like domain with a linker region and the β-propeller domain were analysed separately by SEC-MALS and eluted in peaks with the weight-averaged molar masses of 25.1 kDa for Trx-like domain and 35.7 kDa for the β-propeller. In addition, the purified domains were incubated together and they eluted in a polydisperse peak with the weight-averaged molar mass ranging from 35.2 kDa to 24.8 kDa suggesting that the Trx-like and β-propeller domains do not associate in solution. The ability of the C-terminal domain to associate with the rest of the NHLRC2 molecule was assessed using limited proteolysis of the full-length NHLRC2 by endoproteinase GluC which resulted in the formation of two fragments, one containing the Trx-like and β-propeller domains (aa 1–575) and the other comprising the separate C-terminal domain (aa 587–726). SEC-MALS analysis of the mixture resulted in two separate peaks with weight-averaged masses of 59 kDa (aa 1–575 fragment) and 15 kDa (aa 587–726 fragment), indicating that the C-terminal domain does not stably interact with the rest of the NHLRC2. This domain has been reported to be cleaved off by caspase-8 leading to apoptotic cell death [1]. Altogether, these results demonstrate that all three domains of NHLRC2 do not stably associate with one another in solution.

To obtain an insight into the overall shape of the full-length NHLRC2, we characterized NHLRC2 in solution using SAXS. The MW of 71 kDa derived from Guinier analysis of SAXS scattering curves and related Porod volume is in agreement with sequence-based MW calculation (80.4 kDa) indicating that NHLRC2 exists in a monomeric form in solution. Scattering intensities and normalized Kratky analysis indicate that the full-length NHLRC2 exhibits a compact shape. However, the distance distribution function P(r) profile for the full-length NHLRC2 displays a single asymmetrical peak with a tail, which is indicative of a protein having an elongated shape (S2 Fig). R_g_ and D_max_ obtained from SAXS data (37.54 Å and 146.94 Å, respectively) differ significantly from R_g_ and D_max_ values calculated from the theoretical scattering curve (27.17 Å and 79.62 Å, respectively) generated in CRYSOL for the crystal structure of NHLRC2 (9–572). *Ab initio* shape reconstruction from the experimental SAXS profile using DAMMIF confirms the elongated shape (Fig 2B). The shape of NHLRC2 in solution is more elongated than in the crystal making it difficult to unambiguously dock the crystal structure to the obtained envelope. The elongated shape of the full-length NHLRC2 suggests that the C-terminal β-stranded domain follows the β-propeller and forms a solvent exposed extension. The extension on the N-terminal side of the molecule may represent conformational flexibility of the two α-helices prior to the Trx-like domain. Ensemble optimization method (EOM) [35] analysis with Rflex of 65% shows that the full-length NHLRC2 molecule maintains some degree of internal flexibility consistent with the SEC-MALS data.

### Surface properties

**Fig 3:**
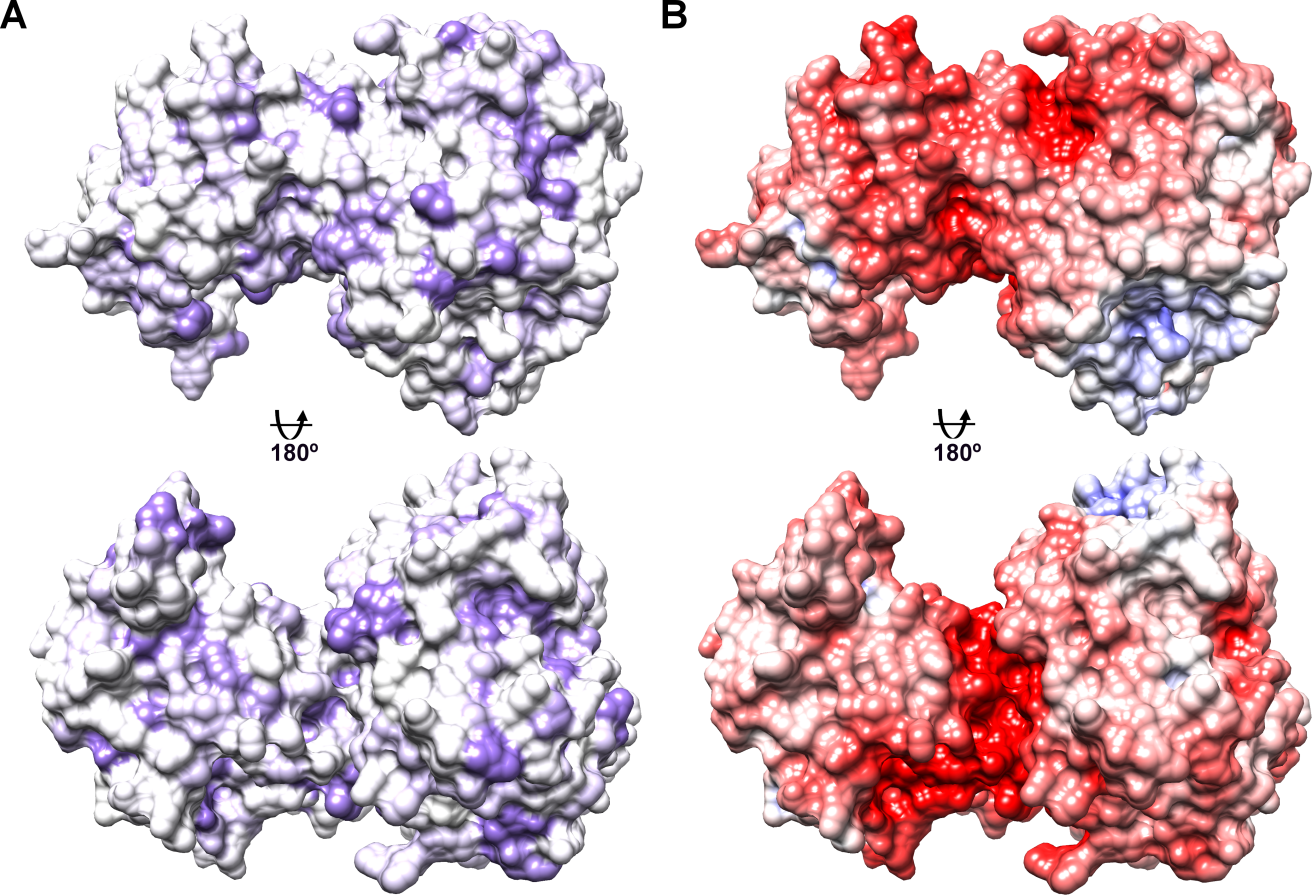
Surface properties of NHLRC2 (9–572). The view is equivalent to that in Fig 1B. (A) Hydrophobic residues are colored in purple. (B) Electrostatic surface potential was calculated using APBS. Negative potential is indicated in red and positive in blue.

To gain an insight into possible regions of NHLRC2 surface that can participate in functional intermolecular interactions, we analysed its surface properties. Analysis of hydrophobic surface reveals numerous small patches of hydrophobic residues scattered across the surface of NHLRC2. However, no dominant hydrophobic area that stands out can be identified (Fig 3A). The electrostatic surface potential was calculated using APBS (Adaptive Poisson-Boltzmann Solver) tool in UCSF Chimera [36]. As expected from the calculated *pI* of the crystallized NHLRC2 fragment, determined by ProtParam to be 5.5, the dominant charge of NHLRC2 surface is negative. However, the electrostatic surface potential analysis also revealed the presence of an extended negative charge running around the surface formed by the faces of two domains suggesting a possible binding site for an interaction partner(s) (Fig 3B).

### Similarity to other structures

NHLRC2 is currently of unknown function. As no structures with a similar domain architecture have been determined previously, we examined whether a structural homology search using the Trx-like and β-propeller domains separately would give any indication towards a possible function.

A DALI search [37] for possible structural homologues using the Trx-like domain returned a list of known crystal structures of Trx8 (or TlpA-like) family members. These included the Trx-like domain of DipZ from *M*. *tuberculosis* (PDB code: 2HYX) [17], DsbF from *M*. *tuberculosis* (PDB code: 1ZZO) [38], DsbE from *P*. *aeruginosa* (PDB code: 3KH7) [39] and thiol-disulfide exchange protein TlpA from *B*. *diazoefficiens* (PDB code: 4TXO) [40] (Panel A in S3 Fig). The Trx-like domain of DipZ was used as a molecular replacement search model and displays the highest sequence identity (28%), compared to other structural homologues. While close structural homologues are all implicated in thiol-disulfide exchange, their active site sequences are distinct from that of NHLRC2. While NHLRC2 has a CCINC motif, structural homologues have SCINC (DipZ), WCPTC (DsbF), WCPSC (DsbE) and WCVPC (TlpA). Consistent with this difference, we have previously shown that the Trx-like domain of NHLRC2 does not contain oxidoreductase activity in a classical insulin reduction assay using either the full-length protein or the isolated Trx-like domain [2] suggesting that it is not involved in thiol-disulfide exchange.

β-propellers are a widespread structural motif of extreme sequence diversity found in numerous proteins with greatly different functions, with the six- and seven-bladed propellers showing the highest degree of functional variation [33]. For example, six-bladed propeller domains are found in enzymes, such as hydrolases and lyases, they are also involved in ligand binding, signaling and maintenance of protein-protein interactions. Depending on the location of a β-propeller relative to other modules in a protein, it can be assigned to different functional groups [33]. The central domain location, like in NHLRC2, is rare and mostly involved in protein-protein interactions. A DALI search [37] for the NHLRC2 β-propeller structural homologues resulted in many hits of very functionally diverse six-bladed propeller-containing proteins, which also displayed very little sequence homology. 26 structural homologues with a Z-score larger than 20 were found by DALI. None of these 26 six-bladed homologues adopts the unique placement of N and C terminal β-strands into separate blades. Among these were three proteins which contained NHL repeats (Panel B in S3 Fig): i) a sensor domain of Ser/Thr kinase PknD from *M*. *tuberculosis* (PDB code: 1RWL, 1.76 Å RMSD across 167 Cα atoms) which has been suggested to switch the activity of kinase domain [41]; ii) the lyase domain of peptidylglycine α-amidating monooxygenase (PAM) from rat (PDB code: 3FVZ, 1.9 Å RMSD across 252 Cα atoms) which catalyzes the second step of amidation reaction of neuropeptides [42]; iii) NHL repeat domain of the Brain Tumor (Brat) protein from *D*. *melanogaster* (PDB code: 1Q7F, 2.2 Å RMSD across 214 Cα atoms) which functions as a translational repressor and a tumor suppressor during development [43]. In addition to the NHL repeat containing proteins, the DALI search returned a large number of common six-bladed propeller enzymes, such as diisopropyl-fluorophosphatase (PDB code: 3U0S) [44], phytase (PDB code: 1CVM) [45], serum paraoxonase (PON) (PDB code: 3SRG) [46], DRP35 from *S*. *aureus*, a bacterial counterpart of eukaryotic PON (PDB code: 2DSO) [47], strictosidine synthase from *R*. *serpentina* (PDB code: 2FP9) [34], and also low-density lipoprotein receptor YWTD domain (PDB code: 1N7D) [48]. Structures of these proteins could be aligned with the NHLRC2 β-propeller with an RMSD between ~2.3 and 2.9 Å while structure-based sequence alignments show only circa 10–18% sequence identities. Structurally homologous six-bladed β-propeller enzymes, except for strictosidine synthase, contain Ca^2+^ or Zn^2+^ ions bound in the central pore which are important for catalytic activity. No electron density corresponding to metal ions were observed in the NHLRC2 β-propeller structure and structure-based alignment does not reveal any relevant residues which might participate in a catalytic reaction. Together this suggests that the domain does not function as an enzyme, but rather that the NHLRC2 β-propeller domain is involved in protein-protein interaction(s). Given that the ‘cup’ of the NHLRC2 β-propeller forms an interface with Trx-like domain, it is plausible to conclude that this surface together with Trx-like domain forms a groove for specific binding of protein ligand(s).

### Bioinformatics analysis of NHLRC2 from different species

Since the crystal structure did not give any definitive answers towards function, we employed a bioinformatics approach to help elucidate a possible function of NHLRC2 or to find potential functional sites in the protein structure. We analysed 3225 Trx-like domain containing sequences of Trx8 family (Trx8-like domain) and narrowed them down to 284 hits containing a CC(I/V)NC active site motif. All of the protein hits had a β-propeller domain after the Trx8-like domain, suggesting potential conservation of function.

**Fig 4:**
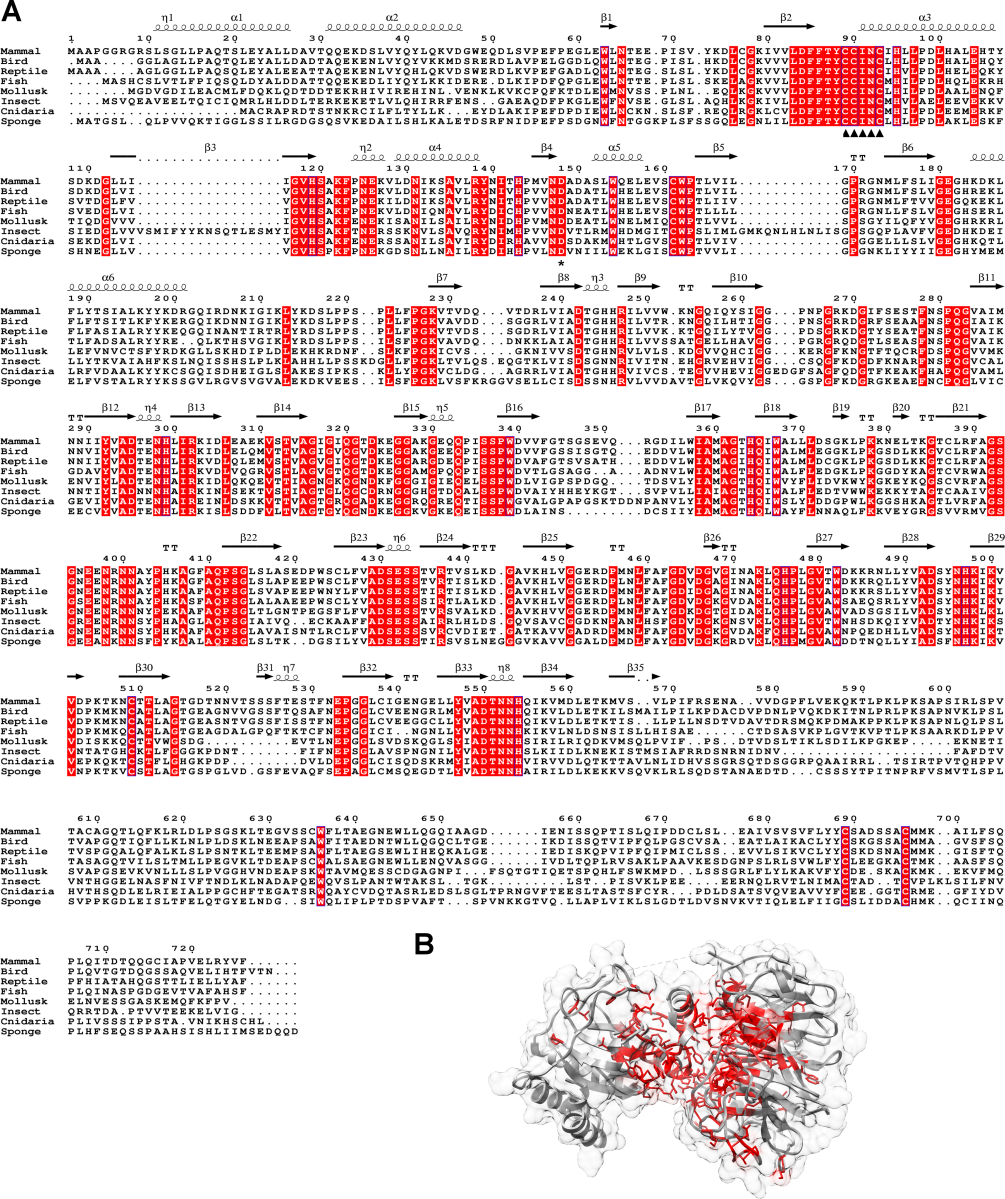
Sequence alignment of NHLRC2 homologs from animalia. (A) The fully conserved residues are highlighted in red. CCINC motif is indicated by arrowheads and Asp-148 by an asterisk. Secondary structure in the alignment is assigned based on the crystal structure. Mammal sequence is equivalent to human NHLRC2 (UniProt ID: Q8NBF2), bird is Zebra finch (UniProt ID: H0ZKU8), reptile is American chameleon (UniProt ID: H9GFX6), fish is Southern platyfish (UniProt ID: M4A111), mollusk is Pacific oyster (UniProt ID: K1Q646), insect is Red fire ant (UniProt ID: E9IZN5), cnidaria is Sea anemone (UniProt ID: A7RH97), sponge (UniProt ID: A0A1X7VMK9). (B) The majority of conserved residues locate to the cleft between the Trx-like and β-propeller domains. The fully conserved residues are mapped on NHLRC2 (9–572) crystal structure and shown in stick representation in red color.

A Blastp [49] search using the human NHLRC2 protein sequence finds homologous sequences from most animalia subdivisions: including mammals, birds, fish, amphibians, reptiles, molluscs, arthropods, cnidarians and sponges, but not from annelids, flatworms or ctenophores. All animalia NHLRC2 homologs maintained a similar domain organization; however, there was virtually no conservation within the C-terminal β-stranded domain (Fig 4A) suggesting that this domain is dispensable for NHLRC2 function or appeared later in evolution.

Eight representative NHLRC2 protein sequences from animalia (Fig 4A) were aligned using Clustal Omega [50] and conserved residues were mapped on NHLRC2 structure (Fig 4B). Consistent with the structure-based analysis, the majority of conserved residues are located mostly in the cleft formed by the Trx-like and β-propeller domain. This suggests that these proteins are orthologs.

**Fig 5:**
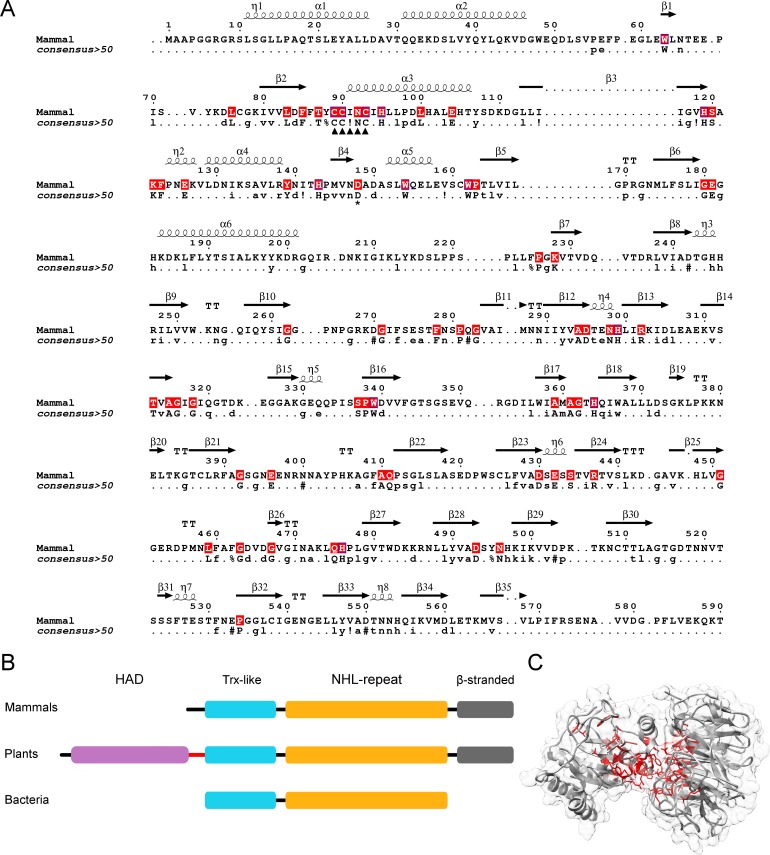
NHLRC2 is present across all kingdoms of life. (A) Trx-like and β-propeller domain sequences from 16 representatives are aligned (the full sequence alignment is in the supplementary material) and conserved residues are indicated in human NHLRC2 (1–590) sequence in red color. The consensus sequence derived from 16 eukaryotic and prokaryotic representatives is provided. CC(I/V)NC motif is indicated by arrowheads and Asp-148 by an asterisk. (B) Domain architecture of NHLRC2 in mammals, plants and bacteria. Plant orthologs contain a transmembrane helix (shown in red) in between HAD and Trx-like domain. Note a small number of bacterial sequences show the mammalian domain architecture. (C) Conserved residues (shown in red) identified in eukaryotic and bacterial orthologs are mapped in human NHLRC2 (9–572) structure.

Previously, NHLRC2 homologs were thought to be limited to animalia [1,2]. However, homologs can be found across different kingdoms of life, including in prokaryotes. Homologs were not found in all species, but rather were scattered. Metazoa, plantae, cyanobacteria, actinobacteria, planctomycetes all had a high density of homologs, while some species of γ-proteobacteria, α-proteobacteria, acidobacteria, chloroflexi, thermotogae, bacteroidetes, firmicutes and amoebozoa also had homologs. Homologs were not found in many prokaryote and some eukaryote clades e.g. no homologs were found in fungi, nor were homologs found in any archaeal species. Homologs clustered into three groups (Fig 5B). Those showing the same domain architecture as NHLRC2, those that lacked the poorly conserved C-terminal β-stranded domain, and homologs, such as those from plants, which contain an additional haloacid dehalogenase-like hydrolase (HAD) domain and a transmembrane helix preceding the Trx8-like domain. Sixteen representative Trx-like and β-propeller domains from eukaryotes and prokaryotes were aligned (Fig 5A) and conserved residues were mapped in the NHLRC2 structure (Fig 5C). Consistent with the analysis of animalia sequences, the conserved residues are located in the cleft formed by the Trx-like and β-propeller domain and include the CC(I/V)NC active site motif of NHLRC2 (Fig 5B). This strongly suggests that these proteins have conserved functions i.e. that they are orthologs.

Since bacterial genomes are often organized into function-linked operons, we examined 7 genomes of distantly related bacterial species which had a NHLRC2 homolog. No conservation was found of adjacent genes, suggesting that this protein is not part of an operon. In one actinobacterium genome the preceding gene contained a HAD domain of the same subtype as found in the plant NHLRC2 homolog, suggesting that the plant protein architecture arose by a gene fusion event.

With the exception of the mammalian and plant homologs we were unable to find reports on functional studies on these proteins. The mammalian protein is essential, its silencing results in embryonic lethality in mice and mutation of NHLRC2 has been associated with severe disease states [2,7], but is of unknown function.

Recently the NHLRC2 ortholog in plant, Suppressor of Quenching (SOQ1), was found to negatively regulate non-photochemical quenching (NPQ) in chloroplasts [51]. In contrast to NHLRC2, SOQ1 is a transmembrane-protein with an N-terminal HAD phosphatase domain on the stromal side of the thylakoid membrane, a transmembrane helix, and the NHLRC2 homologous Trx-like and β-propeller domains on the lumenal side of the thylakoid membrane. The Trx-like and β-propeller domains of SOQ1 share ~40% amino acid identity to the corresponding domains of human NHLRC2 (Panel A in S4 Fig), suggesting that both proteins share a related function. Conservation includes the cleft formed by the Trx-like and β-propeller domain and the active site CCINC (Panel B in S4 Fig). Silencing of SOQ1 in *A*. *thaliana* resulted in weakening of interactions between light-harvesting antenna complexes inducing destabilization of Photosystem II complexes in thylakoid membrane [52]. Additionally, a suppressor screen using Arabidopsis *soq1 npq4* mutant identified the potential downstream target of SOQ1 as plastid lipocalin [53]. In the absence of SOQ1, the electrophoretic mobility of plastid lipocalin changed due to unidentified protein modification. The modification could not be reversed by addition of DTT, strongly indicating that this modification is not linked to thiol-disulfide exchange.

### Analysis of Asp148Tyr found in FINCA patients

**Fig 6:**
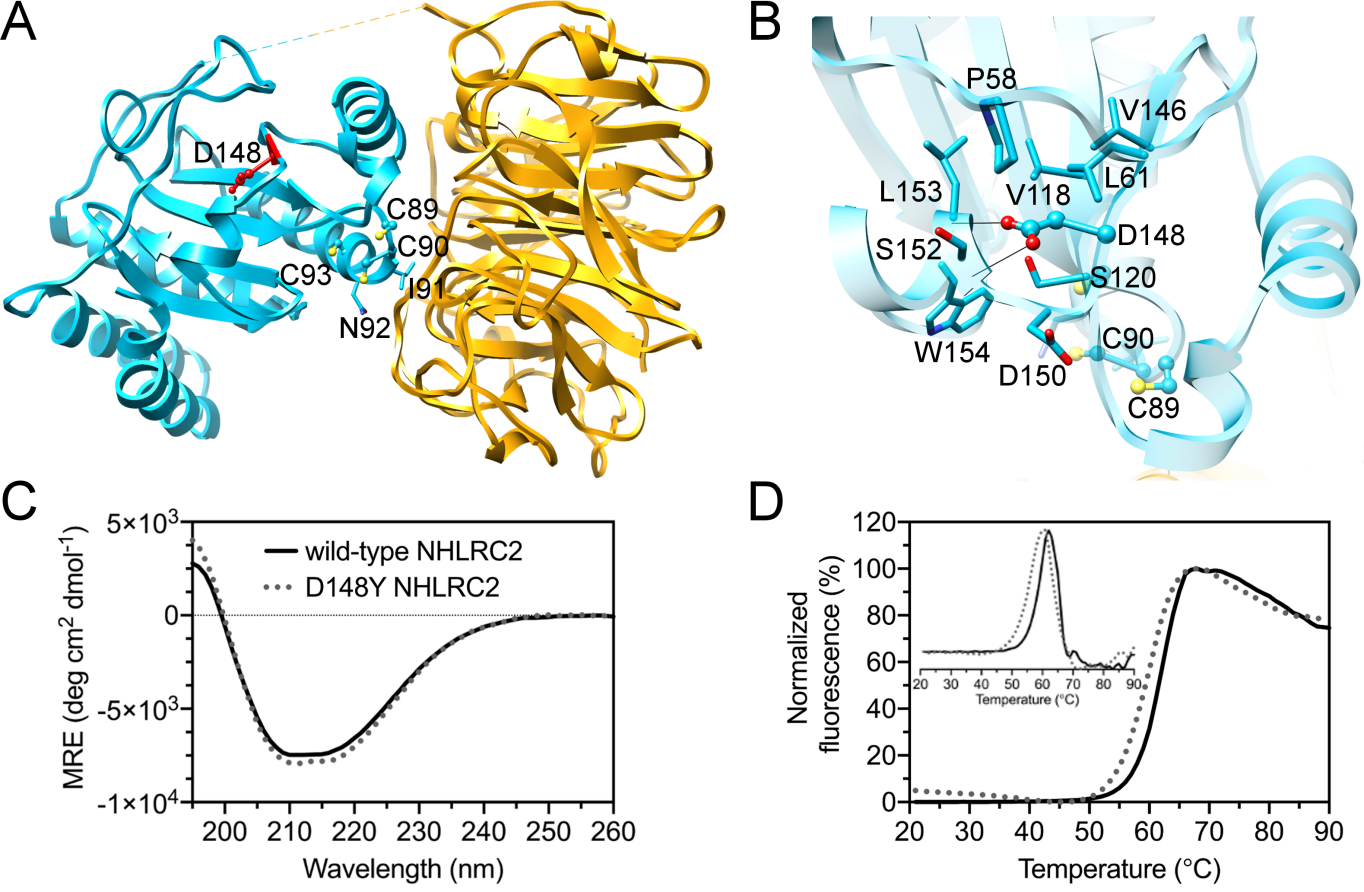
D148Y mutation of NHLRC2 causing FINCA disease. (A) D148 (in red) location in the Trx-like domain of NHLRC2. The CCINC motif is also shown. (B) Close up view of D148 environment in the structure of NHLRC2. The model is colored similarly to that in Fig 1. D148 is shown in ball and stick representation and the neighboring residues are shown in stick representation. Potential hydrogen bonds are shown with black lines. (C) The folding state of the full-length human NHLRC2 and D148Y mutant was assessed by circular dichroism measurements (CD). Visual inspection of the CD spectra indicates that the wild-type NHLRC2 (solid black) and D148Y (grey circles) mutant are folded and contain a mixture of α**-**helices and β-strands. Both spectra are of similar shape indicating a similar amount of regular secondary structure. (D) The thermal stability of the proteins was analyzed by Thermofluor. Wild-type full-length NHLRC2 (solid black) demonstrated a sharp unfolding transition with average T_m_ of 61.5°C and the analysis of derivative showed a cooperative unfolding event. The D148Y mutation (grey circles) resulted in a lower T_m_ of 59.3°C, however, the analysis of derivative still demonstrated a cooperative unfolding, suggesting that the mutation caused small local changes in the protein structure and stability.

A mutation in human NHLRC2 Asp-148 to Tyr was identified in a recent study by Uusimaa [2] and has been associated with a novel lethal disease FINCA. Asp-148 is found in the homologous protein from many species, but it is not fully conserved. Asp-148 is located in the Trx-like domain, between β-strand β3 and α-helix α6 (Fig 6A). Asp-148 interacts via hydrogen bonds with back bone amides of Ser-152 and Leu-153 (Fig 6B). The introduction of the larger Tyr residue may disrupt hydrogen bonding and destabilize the conformation of the loop and the α-helix. Analysis of the mutated full-length NHLRC2 by CD did not reveal any changes in the secondary structure and Thermofluor assay showed a 2°C reduction in the melting temperature (Fig 6C and 6D). In contrast to the wild-type protein, we could not crystallize NHLRC2 (1–572) containing D148Y mutation despite extensive crystallization screening and MMS experiments. This *in vitro* data, taken together with the *in vivo* data that the D148Y mutation results in a large decrease of NHLRC2 levels in cells derived from patients [2], suggests that the mutation may destabilize the overall structure of NHLRC2.

## Conclusions

The core structure of NHLRC2 appears in some animals, plants and unicellular eukaryotes and prokaryotes. The Trx-like and β-propeller domains are conserved; and residues located in the cleft formed by the Trx-like and β-propeller domain, including the Trx-like active site motif of NHLRC2, show a high degree of conservation. This strongly suggests that these are orthologs. The protein is essential in mammals and modulates the light harvesting efficiency in plants. Data from the plant ortholog suggests that it is involved in an as yet unidentified protein modification. What this modification is and why orthologs can be found in a scattering of prokaryotic and eukaryotic species, but not in others remains to be identified.

## Supporting information

S1 Fig Internal symmetry of the NHLRC2 β-propeller. (A) Structural superimposition of the six blades. Strand A and D are indicated. Each blade fragment starts from the cup residues (top) and the first three β-strands (A–C) superimpose well. The backbone RMSD between all six blades ~0.8 Å. (B) Structure-based alignment of the six blades. Blade numbers are listed on the right, residues numbers are listed on the left. Each strand is indicated and the XXX#§ motif is depicted. Conserved structural prolines and glycines are marked by asterisk and are indicated by arrows on panel (A).
(TIF)

S2 Fig SAXS analysis of the full-length NHLRC2. (A) Raw SAXS data collected for concentration series (5 mg/ml in green, 2.5 mg/ml in blue and 1.25 mg/ml in purple) overlaid with theoretical scattering curve calculated from NHLRC2 (9–572) crystal structure using *CRYSOL* (in red). Guinier regions for each dataset are shown in the inset. (B) R_g_ and I(0)/c plotted against concentration. (C) Kratky plot generated from the SAXS data collected at 5 mg/ml. (D) Distance distribution plot.
(TIF)

S3 Fig Superimposition of NHLRC2. Trx-like domain (A) and β-propeller domain (B) with structural homologs identified by DALI search. Structural homologs are indicated with the corresponding PDB code. DipZ from *M*. *tuberculosis* (2HYX; [17]), DsbF from *M*. *tuberculosis* (1ZZO; [[38]), DsbE from *P*. *aeruginosa* (3KH7; [39]), thiol-disulfide exchange protein TlpA from *B*. *diazoefficiens* (4TXO; [40]), a sensor domain of Ser/Thr kinase PknD from *M*. *tuberculosis* (1RWL; [41]), the lyase domain of peptidylglycine α-amidating monooxygenase (PAM) from rat (3FVZ; [42]), NHL repeat domain of the Brain Tumor (Brat) protein from *D*. *melanogaster* (1Q7F; [43]).
(TIF)

S4 Fig Comparison of NHLRC2 with SOQ1. (A) Sequence alignment. Conserved residues are marked in red color. CCINC motif is indicated by arrowheads and Asp-148 by asterisk. (B) Conserved residues are indicated on NHLRC2 (9–572) structure and shown in stick representation in red color.
(TIF)
