# Supplementary figures and images for: Structural analysis of human NHLRC2, mutations of which are associated with FINCA disease
Source: PLoS One. 2018 Aug 23;13(8):e0202391. doi: 10.1371/journal.pone.0202391 (PMC6107167; doi:10.1371/journal.pone.0202391)

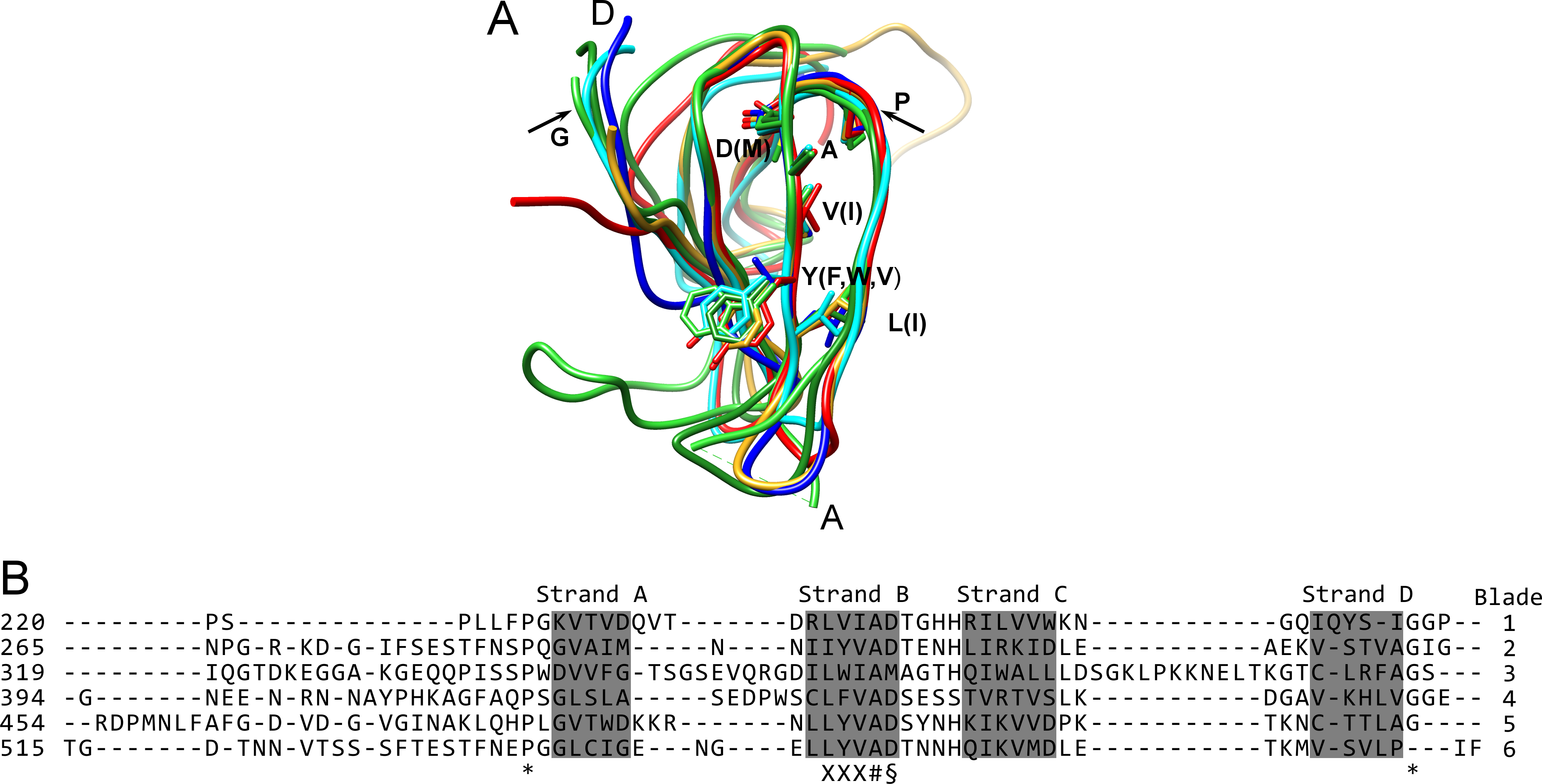

Supplement: S1 Fig — (A) Structural superimposition of the six blades. Strand A and D are indicated. Each blade fragment starts from the cup residues (top) and the first three β-strands (A–C) superimpose well. The backbone RMSD between all six blades ~0.8 Å. (B) Structure-based alignment of the six blades. Blade numbers are listed on the right, residues numbers are listed on the left. Each strand is indicated and the XXX#§ motif is depicted. Conserved structural prolines and glycines are marked by asterisk and are indicated by arrows on panel (A). (TIF) [file pone.0202391.s001.tif]

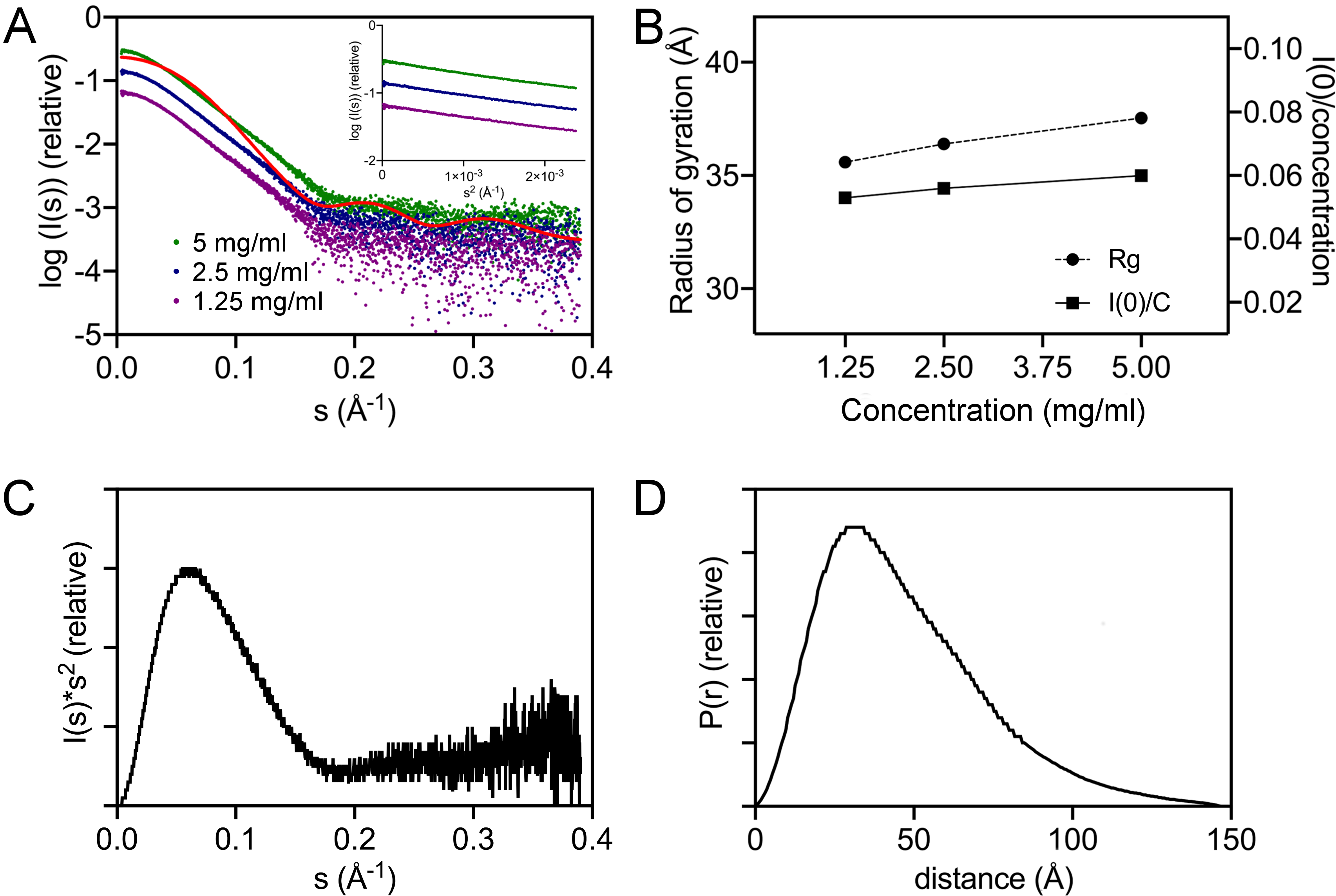

Supplement: S2 Fig — (A) Raw SAXS data collected for concentration series (5 mg/ml in green, 2.5 mg/ml in blue and 1.25 mg/ml in purple) overlaid with theoretical scattering curve calculated from NHLRC2 (9–572) crystal structure using CRYSOL (in red). Guinier regions for each dataset are shown in the inset. (B) Rg and I(0)/c plotted against concentration. (C) Kratky plot generated from the SAXS data collected at 5 mg/ml. (D) Distance distribution plot. (TIF) [file pone.0202391.s002.tif]

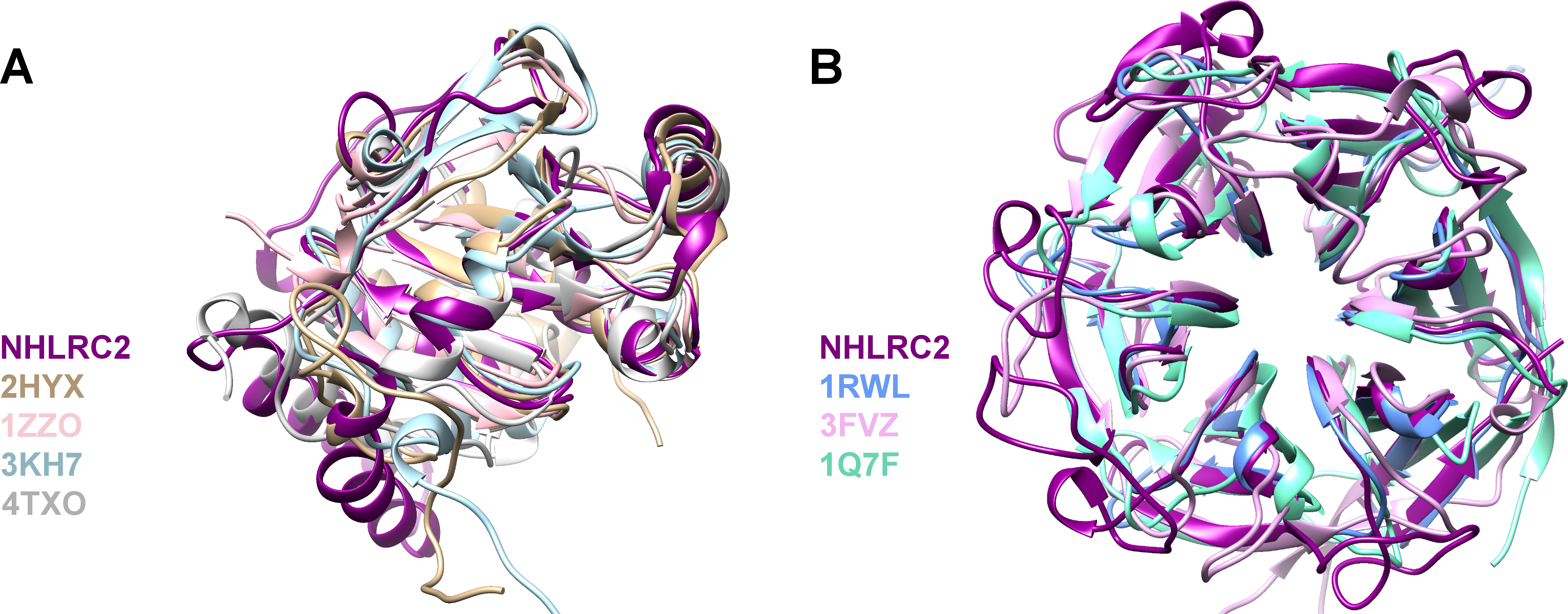

Supplement: S3 Fig — Trx-like domain (A) and β-propeller domain (B) with structural homologs identified by DALI search. Structural homologs are indicated with the corresponding PDB code. DipZ from M. tuberculosis (2HYX; [17]), DsbF from M. tuberculosis (1ZZO; [[38]), DsbE from P. aeruginosa (3KH7; [39]), thiol-disulfide exchange protein TlpA from B. diazoefficiens (4TXO; [40]), a sensor domain of Ser/Thr kinase PknD from M. tuberculosis (1RWL; [41]), the lyase domain of peptidylglycine α-amidating monooxygenase (PAM) from rat (3FVZ; [42]), NHL repeat domain of the Brain Tumor (Brat) protein from D. melanogaster (1Q7F; [43]). (TIF) [file pone.0202391.s003.tif]

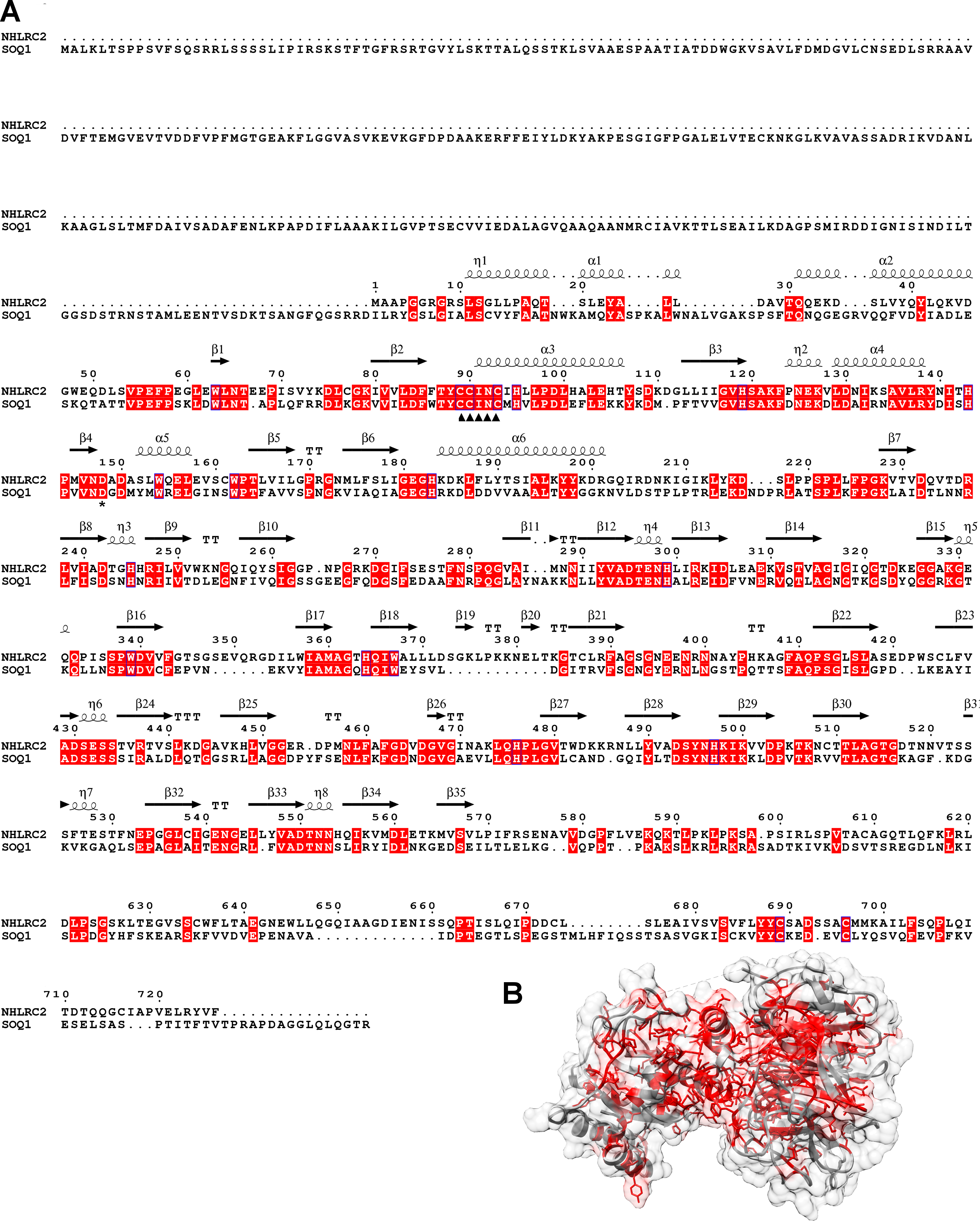

Supplement: S4 Fig — (A) Sequence alignment. Conserved residues are marked in red color. CCINC motif is indicated by arrowheads and Asp-148 by asterisk. (B) Conserved residues are indicated on NHLRC2 (9–572) structure and shown in stick representation in red color. (TIF) [file pone.0202391.s004.tif]
